# Inhibition of human kynurenine aminotransferase isozymes by estrogen and its derivatives

**DOI:** 10.1038/s41598-017-17979-7

**Published:** 2017-12-14

**Authors:** Gayan S. Jayawickrama, Alireza Nematollahi, Guanchen Sun, Mark D. Gorrell, W. Bret Church

**Affiliations:** 1 Group in Biomolecular Structure and Informatics, Faculty of Pharmacy, The University of Sydney, Sydney, NSW 2006 Australia; 2 Molecular Hepatology Laboratory, Centenary Institute and Sydney Medical School, The University of Sydney, Sydney, NSW 2006 Australia

## Abstract

The kynurenine aminotransferase (KAT) enzymes are pyridoxal 5′-phosphate-dependent homodimers that catalyse the irreversible transamination of kynurenine into kynurenic acid (KYNA) in the tryptophan metabolic pathway. Kynurenic acid is implicated in cognitive diseases such as schizophrenia, and several inhibitors have been reported that selectively target KAT-II as it is primarily responsible for kynurenic acid production in the human brain. Not only is schizophrenia a sexually dimorphic condition, but women that have schizophrenia have reduced estrogen levels in their serum. Estrogens are also known to interact in the kynurenine pathway therefore exploring these interactions can yield a better understanding of the condition and improve approaches in ameliorating its effects. Enzyme inhibitory assays and binding studies showed that estradiol disulfate is a strong inhibitor of KAT-I and KAT-II (IC_50_: 291.5 μM and 26.3 μM, respectively), with estradiol, estradiol 3-sulfate and estrone sulfate being much weaker (IC_50_ > 2 mM). Therefore it is possible that estrogen levels can dictate the balance of kynurenic acid in the brain. Inhibition assay results and modelling suggests that the 17-sulfate moiety in estradiol disulfate is very important in improving its potency as an inhibitor, increasing the inhibition by approximately 10–100 fold compared to estradiol.

## Introduction

Schizophrenia has a prevalence of approximately 1% worldwide^1,2^, and is a major societal and individual health burden owing to the debilitating nature of the positive symptoms (such as hallucinations, delusions), negative symptoms (such as social withdrawal, flattened affect), and cognitive dysfunction that is associated with this condition^3^. Sexual dimorphism has been described for the age of onset of schizophrenia in several studies^4–6^. Males typically have been shown to have an earlier onset, with a peak in those aged 15–25 years^6^. In comparison, the onset for females peak in the ages of 20–29 years^6^. The relatively lower incidence of schizophrenia in females during adolescence corresponds to a time of major hormonal changes, including that of increasing estrogen levels^7^. A smaller secondary peak for late onset schizophrenia has also been observed in females aged 45–49 years^6^ which again coincides with a period of estrogen change in women, with this time it being a drop in estrogen levels during menopausal transition^8,9^. The association of estrogen deficits in schizophrenia has been supported by molecular, animal and clinical studies. Several studies have identified increased severity of schizophrenia or surrogate measures of schizophrenia associated with low circulating estrogen levels^10,11^. In women with schizophrenia, reduced levels of serum estradiol has been reported in all phases of their menstrual cycle and although some reduction in estrogen is known to be associated with some antipsychotic medications, for which mechanisms leading to hypoestrogenism are known, it is thought that the reduction in women with schizophrenia exists independently of medication^10,11^.

**Figure 1:**
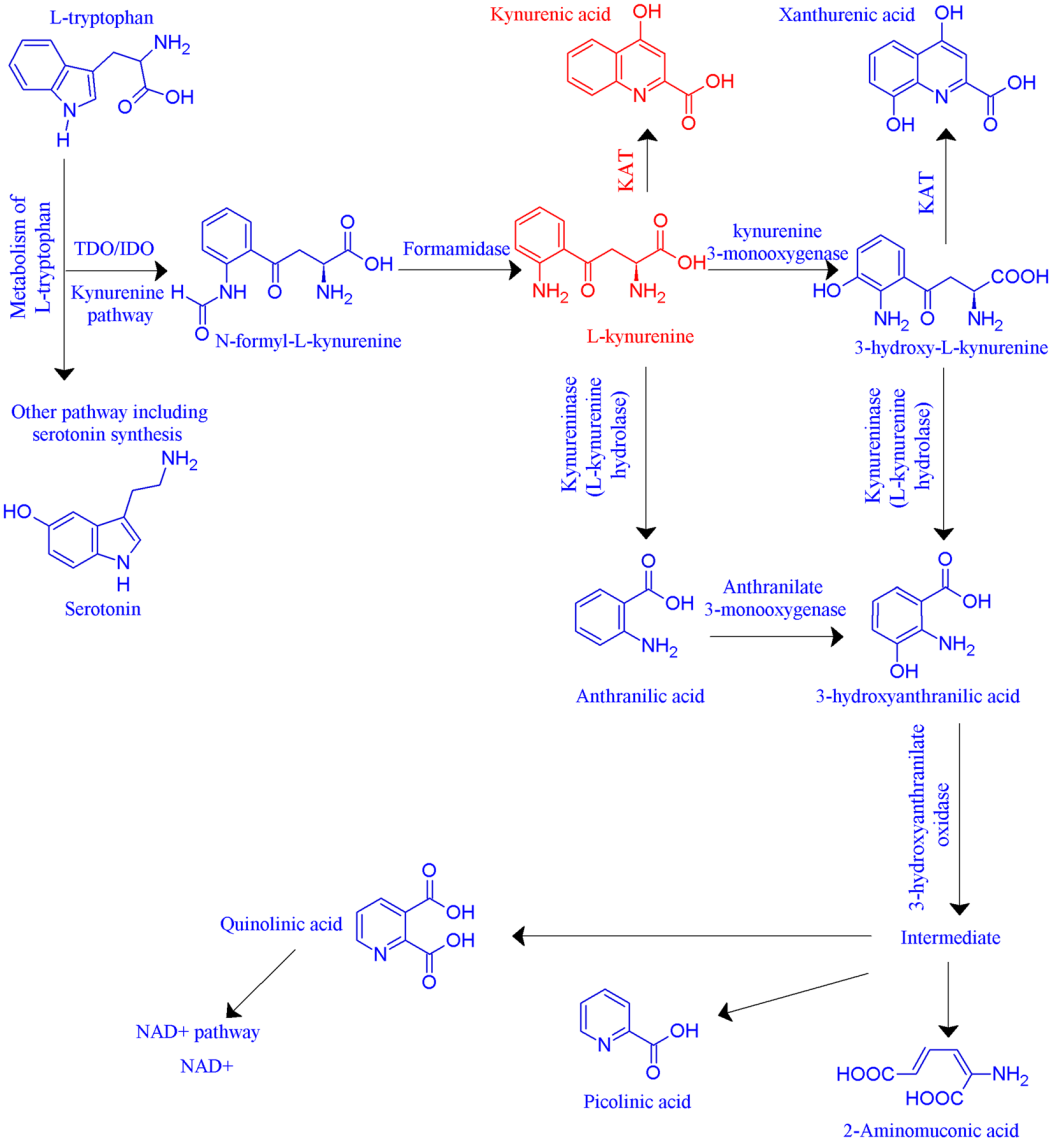
The kynurenine pathway. The first step is rate-limiting, involving tryptophan getting cleaved by indoleamine 2,3-dioxygenase (IDO1/IDO2; EC 1.13.11.52) or tryptophan 2,3-dioxygenase 2 (TDO2; EC 1.13.11.11) to form N-formylkynurenine. Kynurenine formamidase (EC 3.5.1.9) metabolises this further into L-kynurenine, where it is converted into either kynurenic acid by kynurenine aminotransferases (KAT; EC 2.6.1.7), 3-hydroxykynurenine (3-HK) by kynurenine 3-monooxygenase (EC 1.14.13.9), or anthranilic acid by kynureninase (EC 3.7.1.3). 3-HK can be metabolised into xanthurenic acid by KAT, or 3-hydroxyanthranilic acid (3-HANA) by kynureninase. The latter is also a product that is formed by anthranilate 3-monooxygenase (EC 1.14.16.3) acting on anthranilic acid. Downstream of 3-HANA, quinolinic acid is formed and this progresses into nicotinamide metabolism which produces nicotinamide adenosine dinucleotide (NAD). The transamination of kynurenine to kynurenic acid by the KAT enzymes is denoted in red. Figure adapted with permission from *Jayawickrama*, *et al*.^24^.

The estrogen hormones primarily play an important role in growth and development, however they also display additional functions including influencing the breakdown of tryptophan. Tryptophan is an essential amino acid that must be acquired through the diet. In its unbound form, tryptophan is able to cross the blood brain barrier^12^ where it is a precursor for the serotonin pathway and the kynurenine pathway (Fig. 1).

**Figure 2:**
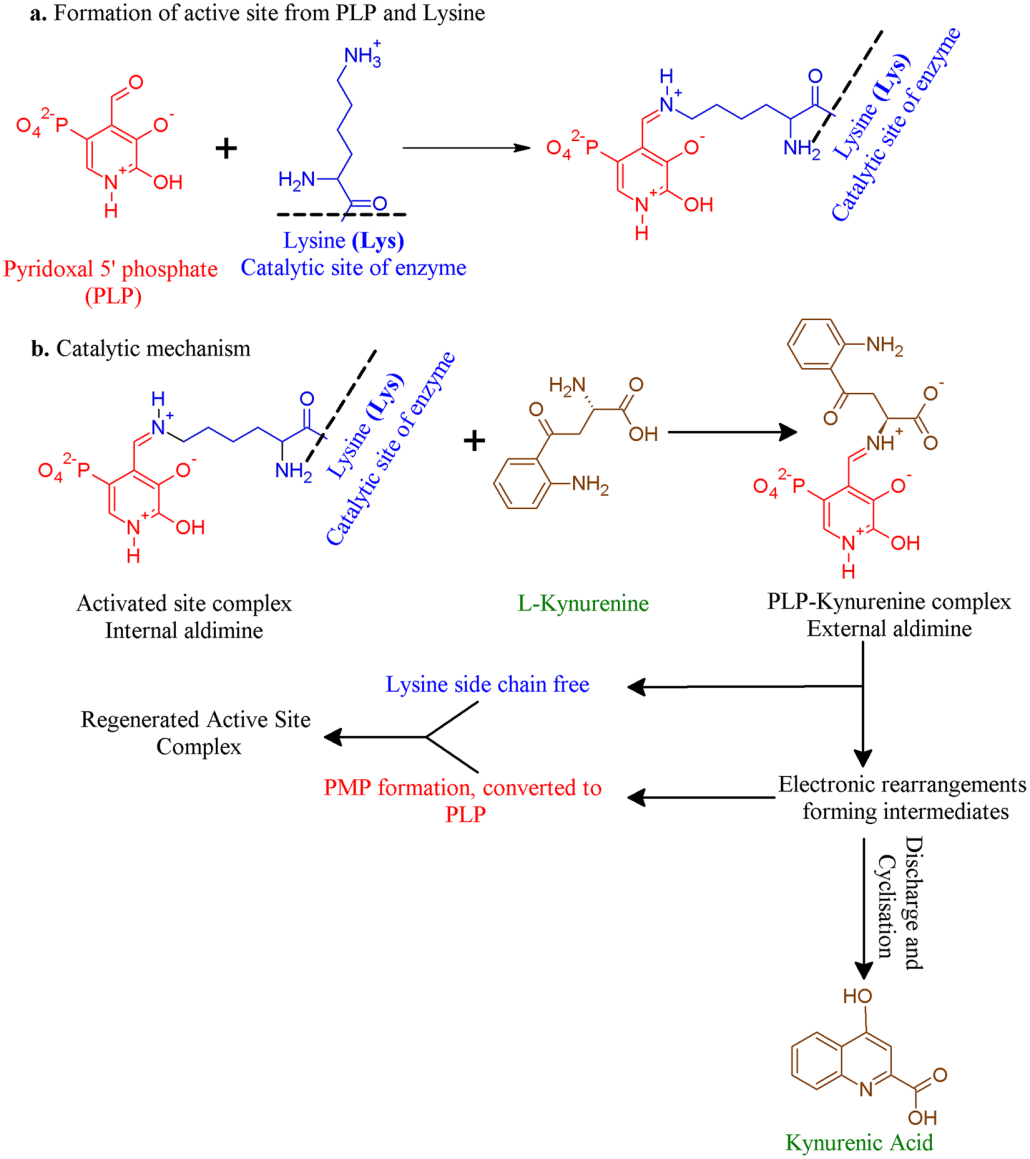
PLP-dependent transamination reaction. (**a**) The active site is formed from PLP (red) and Lys-263 (blue). (**b**) The transamination process which irreversibly converts kynurenine (brown) into kynurenic acid (brown); PLP is regenerated in this process via an α-ketoacid acceptor. Figure adapted with permission from *Jayawickrama*, *et al*.^24^.

Up to 99% dietary tryptophan may be metabolised through the complex kynurenine pathway^13^. This pathway includes a family of pyridoxal 5′-phosphate (PLP)-dependent enzymes called kynurenine aminotransferase (KAT)^14^, of which there are four KAT isoforms in mammals. Between them, they are responsible for the irreversible transamination of kynurenine (KYN) to kynurenic acid (KYNA), using PLP as a cofactor (Fig. 2)^15^. The KAT enzymes are homodimers and each subunit includes an N-terminal arm, a large domain containing the PLP-binding site, and a small domain containing the C-terminus^14,16^.

**Figure 3:**
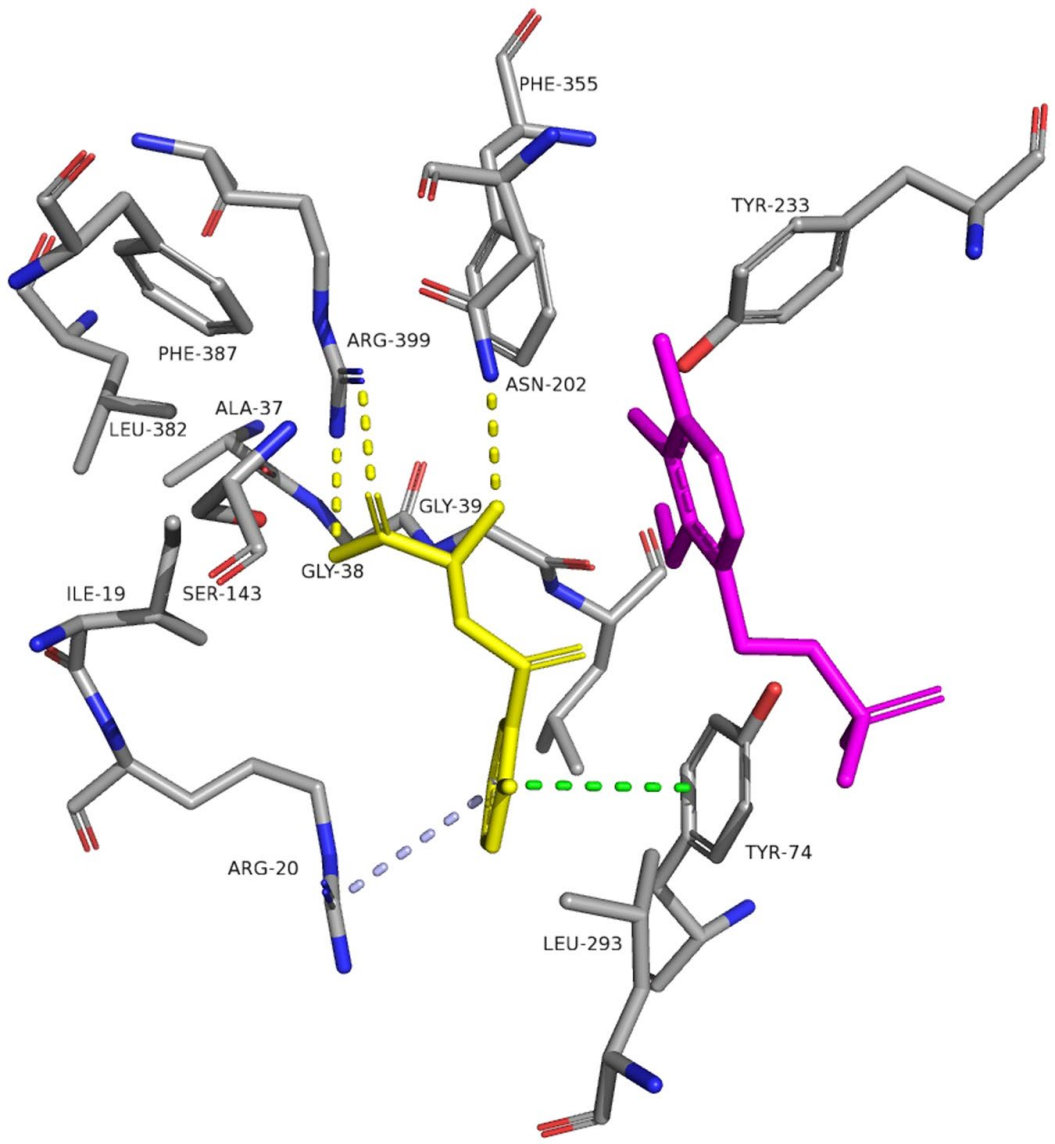
Kynurenine in the active site of KAT-II. The amino acids with an atom within 5.0 Å of kynurenine (yellow), and PLP (magenta), were chosen for display. Residue Tyr-142 was removed for clarity. The aromatic ring of kynurenine forms pi-pi interactions (green dashes) with Tyr-74, and pi-cation (blue dashes) interactions with Arg-20. The carboxyl moiety forms a salt bridge (yellow dashes) with Arg-399, and the neighbouring amine group forms hydrogen bonds with Asn-202 (yellow dashes). Image generated with PyMOL^67^.

Examination of the structural details of the transamination mechanism of kynurenine with KAT-II identifies the significance of some regions (Fig. 3). The side-chain of Arg-20 has a π-cation interaction with the aromatic ring of kynurenine, which is the location around which there is a conformational fold of the N-terminal region residues and therefore seemingly controls the entry point of the substrate into the active site^15^. Arg-399 acts as a salt bridge and interacts with the carboxyl of the kynurenine thereby anchoring the neighbouring amine group of the substrate in the active site, and this is also assisted by the side chains of Asn-202 and Gly-39 forming hydrogen bonds with the amino acid group. Lys-263 is important in the transamination process as it is responsible for situating PLP by an internal aldimine linkage, which is later broken during the transamination process^15^.

KYNA, the product of the transamination, is an N-methyl-D-aspartic acid (NMDA)^17,18^ and an α7-nicotinic acetylcholine receptor antagonist^19^ that plays a role in cognitive function. KYNA has been shown to have anticonvulsive properties^20,21^ and this, alongside its antagonistic activity of the excitatory NMDA receptor, allows KYNA to be considered neuroprotective. However, KYNA has also been found elevated in the cerebrospinal fluid^22^ and prefrontal cortex^23^ of patients with schizophrenia. Therefore, among the contributing aetiologies on the pathogenesis of schizophrenia, which includes the dopamine, GABAergic and glutamatergic hypotheses^24^, KYNA has also been implicated. Developing KAT inhibitors, to reduce KYNA levels, has been pursued as a potential means of ameliorating the effects of schizophrenia^25^.

**Figure 4:**
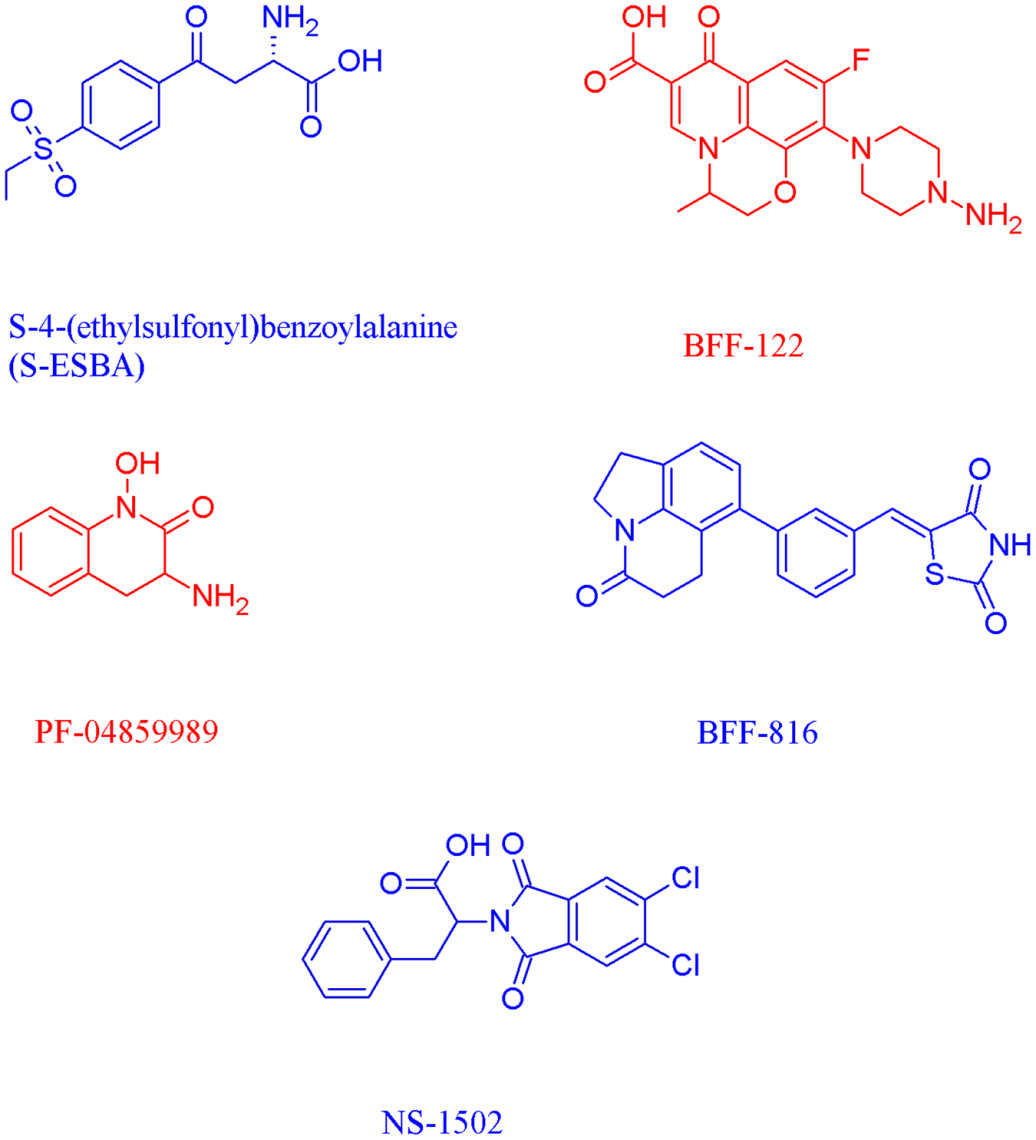
Chemical structures of KAT-II inhibitors S-ESBA, BFF-122, PF-04859989, BFF-816, and NS-1502. The reversible inhibitors are drawn in blue; the irreversible inhibitors are in red.

S-ESBA, BFF-122, PF-04859989, BFF-816 (Fig. 4) are KAT-II inhibitors that have demonstrated a proficiency in reducing KYNA levels in rat homogenates and dialysates^26–29^, and often simultaneously increase the levels of the key neurotransmitters, glutamate^29,30^, acetylcholine^31^, dopamine^32^, and GABA^33^, which have recognized roles in cognitive function^18^. Supplementing this, the use of S-ESBA, BFF-816 and PF-04859989 on rats and, in the case of the latter inhibitor, also on non-human primates, have shown that these inhibitors may improve memory and spatial learning^29,30,34^.

All these inhibitors referred to, and the recently synthesised NS-1502^35^ (Fig. 4), have been designed to selectively target the KAT-II isoform as it is thought to be the main KAT that is responsible for synthesising KYNA in the brain^36^. While very potent, BFF-122 and PF-04859989 were found to act through an irreversible inhibitory mechanism by forming a covalent adduct with PLP^28,37^. However the later inhibitors, BFF-816 and NS-1502, pursued reversibility in their design, which may be preferable due to the ubiquitous nature of PLP-dependent enzymes in the body. Over 300 PLP-dependent enzymes exist and potentially deactivating these could cause adverse effects, as has been seen previously with the development of dyskinesias and increasing death rates in patients with Parkinson’s disease following the introduction of carbidopa into their medication regimen^38,39^.

Steroid compounds, such as estrogens, and their sulfate and phosphate esters forms, have activity on various PLP-dependent enzymes^40,41^, including those in the kynurenine pathway. In rat kidney and liver homogenates, they were shown to reversibly inhibit kynureninase^42,43^, kynurenine monooxygenase^44^, and KAT^40,45^, and induce tryptophan 2,3-dioxygenase 2 (TDO2)^46–48^, although other evidence refutes TDO2 induction^49^. This discrepancy might arise from inhibition of enzymes such as kynureninase and KAT by estrogens, thereby causing a build-up of kynurenine. As kynurenine is the measured, downstream product of TDO2 activity, a build-up of kynurenine can potentially give an appearance of TDO2 induction in a tissue homogenate.

The effects of estrogen on the kynurenine pathway metabolites have been observed in women. Relative to controls, young women taking oral contraceptives have increased excretion of kynurenine pathway metabolites, including kynurenine, xanthurenic acid, 3-hydroxykynurenine, 3-hydroxyanthranilic acid and quinolinic acid, after they were given a tryptophan load^50–54^. These changes are caused by the estrogen component in the oral contraceptives, as women administered progestogen alone do not display increased excretion of these metabolites^55,56^. Similarly, elevated metabolite excretory patterns were displayed during pregnancy^57^ and in ovulation in the menstrual cycle^58^, both correlating with rising estrogen levels.

**Figure 5:**
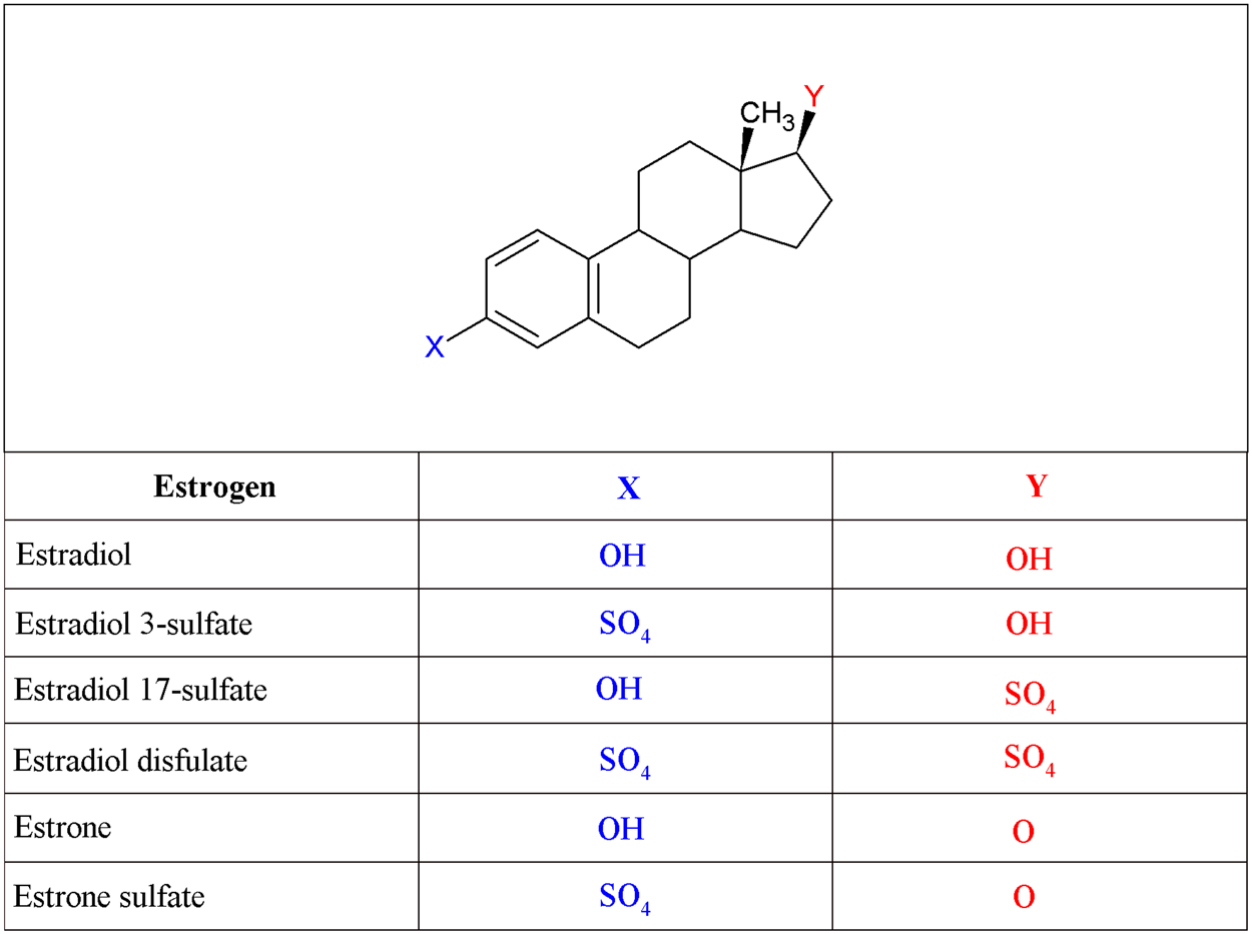
Scaffold of estrogen compounds. X is off the 3-carbon position. Y is off the 17-carbon position. The four rings are labelled A, B, C, D, respectively.

Owing to the sexual dimorphism in schizophrenia and the observed links between the kynurenine pathway and schizophrenia, exploring the relationship between estrogen and the kynurenine pathway will aid in the molecular understanding of this pathway and its associated conditions in which variations occur. In our current study, our goal was to accurately establish the inhibitory capacity of estradiol, estradiol 3-sulfate, estradiol disulfate and estrone sulfate (Fig. 5) directly on recombinant human KAT-I and KAT-II. By means of surface plasmon resonance (SPR) and modelling we were able to further characterise the probable binding mechanics of the important estrogen metabolites. A detailed study of the binding will aid in the drug design process of KAT-II inhibitors.

## Results and Discussion

### HPLC Inhibition Assay of Estrogens on KAT-I and KAT-II

**Figure 6:**
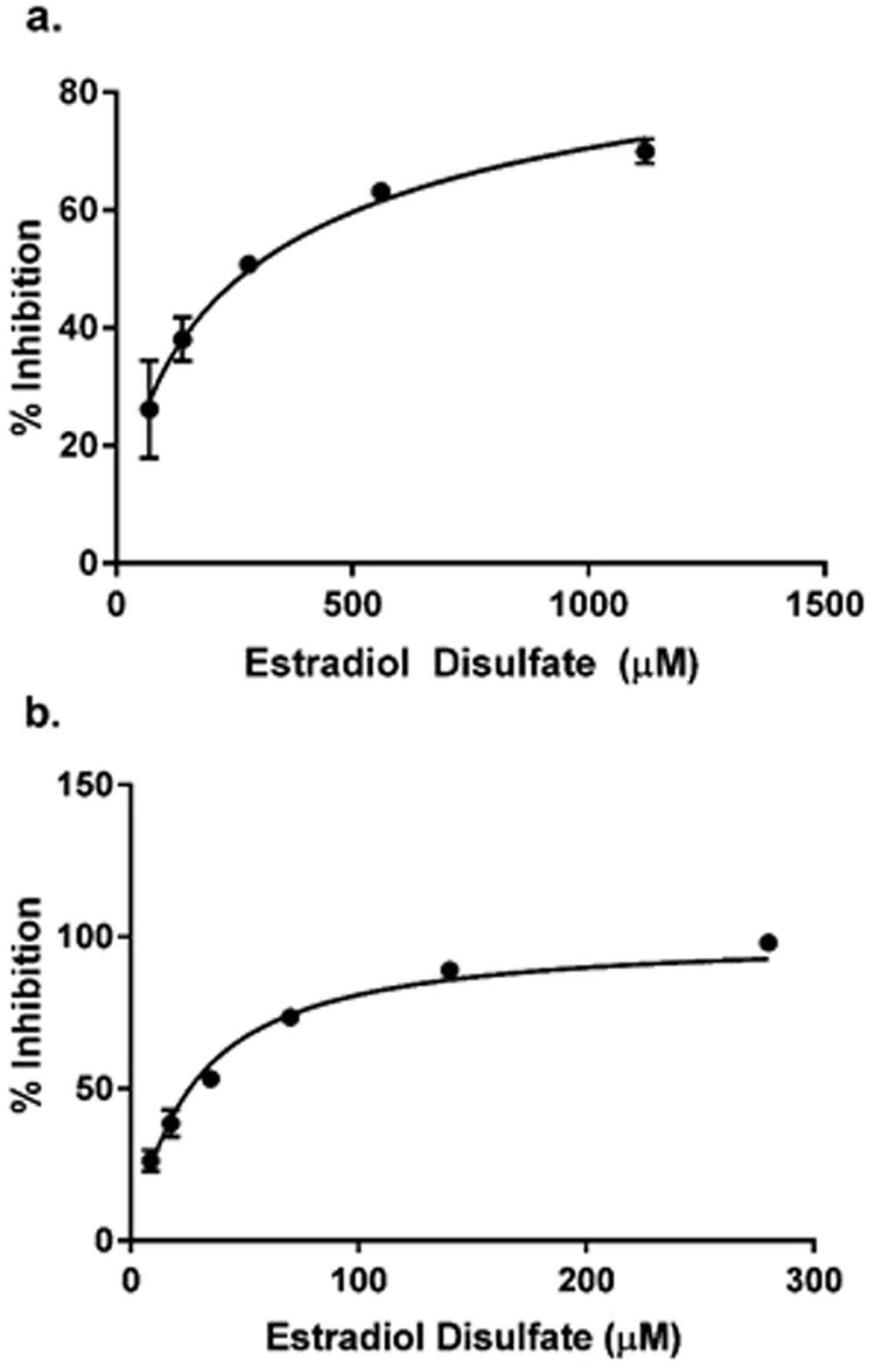
Inhibition assays of estradiol disulfate on KAT. (**a**) Inhibitory activity of estradiol disulfate in a dose-dependent format on KAT-I (IC_50_: 291.5 (±19.6) μM, R^2^ = 0.95). (**b**) Inhibitory activity of estradiol disulfate in a dose-dependent format on KAT-II (IC_50_: 26.3 (±1.4) μM, R^2^ = 0.98). Both plots used data from at least 3 sets of tests. Diagrams were produced using GraphPad Prism v7.02^59^.

Estradiol, estradiol 3-sulfate and estrone sulfate were found to not be good inhibitors of human KAT-I at the concentrations used (IC_50_: >2 mM). Estradiol disulfate displayed relatively good inhibitory capacity and had an IC_50_ of 291.5 (±19.6) μM (Fig. 6a).

Similar to KAT-I, Estradiol and estradiol 3-sulfate were not very good inhibitors of human KAT-II (IC_50_: >2 mM). Estrone sulfate also could not be fully profiled and hence its IC_50_ is approximately ~2 mM. Estradiol disulfate however was found to inhibit KAT-II comparatively well, with an IC_50_ of 26.3 (±1.4) μM (Fig. 6b), and also it was determined that this inhibition is reversible. This was demonstrated by varying the concentration of PLP in the reaction mixture which resulted in the inhibition caused by estradiol disulfate reducing as PLP concentration increased, representing competition with it. This contrasts with irreversible inhibitors, such as PF-04859989, where the inhibitory activity remains unchanged with varying PLP cβoncentrations (see Supplementary Table S1).

Previously the inhibition profiles of the known reversible inhibitor NS-1502 (IC_50_: 315 μM), and irreversible inhibitors BFF-122 (IC_50_: 15–20 μM) and PF-04859989 (1–3 μM) on KAT-II have been measured^35^. Our IC_50_ for the reversible estradiol disulfate suggests that it inhibits KAT-II strongly by the standards of the previously reported inhibitors, as it compares in magnitude to the irreversible inhibitor, BFF-122.

Our results indicate that the disulfate ester of estradiol had a strong, reversible, inhibitory capacity for both KAT-I and KAT-II, while estrone sulfate was a weak inhibitor of KAT-II only. Relatively, estradiol and estradiol 3-sulfate did not inhibit either enzyme significantly. These results suggest that having a sulfate moiety on the 17-position of estradiol could be important for inhibitory potency. The addition of a 3-sulfate group onto estradiol did not alter inhibitory potency whereas estradiol disulfate, which additionally contains a 17-sulfate group, displayed markedly improved inhibition. However, the importance of the 17-sulfate can only be inferred as estradiol 17-sulfate was not tested directly.

### Surface Plasmon Resonance Analysis of Estradiol Disulfate on KAT-II

SPR was employed to evaluate the binding profile of estradiol disulfate on KAT-II. KAT-II was bound to a CM5 chip by amine coupling (achieving approximately 8000 RU). Estradiol disulfate was injected at increasing concentrations (170 nM–43750 nM) and the corrected, reference subtracted data displayed that estradiol disulfate interacted with KAT-II in a dose-dependent manner, and it had rapid association and dissociation with KAT-II. Due to the nature of this interaction, kinetic analyses could not be performed to directly evaluate the association and dissociation rate constants. Instead, a steady state affinity model was fitted with this data which showed that estradiol disulfate binds with a KD of 5.05 × 10^−6^ (chi^2^: 0.481) (see Supplementary Fig. S1), comparable to SPR measurements of NS-1502 interacting with KAT-II (KD: 7.2 × 10^−6^, chi^2^: 0.34)^35^. The KD suggests that estradiol disulfate binds with affinity as strong as that would be expected from a compound that is neither irreversible nor very tightly binding.

### Computational Docking of Estrogen Compounds on KAT-II

Computational docking showed that estradiol disulfate displays the best potential for affinity with KAT-II at the active site, with a G score of −8.0 Kcal/mol (see Supplementary Table S2). Estradiol is oriented with the aromatic, A ring forming a pi-cation interaction with Arg-20, while the C and D rings project towards Arg-399 and Asn-202, with the 17-hydroxyl hydrogen bonding with Arg-399. (see Supplementary Fig. S2). This pose mirrors the interactions of the natural substrate, kynurenine, and KAT-II, where the aromatic ring in kynurenine has an analogous interaction with Arg-20 and the carboxyl forms a salt bridge with Arg-399 and helps orientate it in the active site^15^. By binding similarly, estradiol is likely to directly compete in the active site with the substrate and cause some competitive inhibition. This was seen in the inhibition assay results, but ultimately the inhibition by estradiol was relatively weak.

Estrone has a similar pose, with the 17-ketone group hydrogen bonding with the Arg-399 and Asn-202 side-chains (see Supplementary Fig. S3). Like Arg-399, Asn-202 is typically involved in orientating kynurenine in the active site by forming hydrogen bonds with the amino acid group in kynurenine^15^. Estradiol 3-sulfate (see Supplementary Fig. S4) and estrone sulfate (see Supplementary Fig. S5) are also similarly situated, with the sulfate at the 3-position forming hydrogen bonds with the Arg-20. As with estradiol, these poses reflect the way in which kynurenine interacts with the active site but the compounds themselves were not strongly inhibitory.

**Figure 7:**
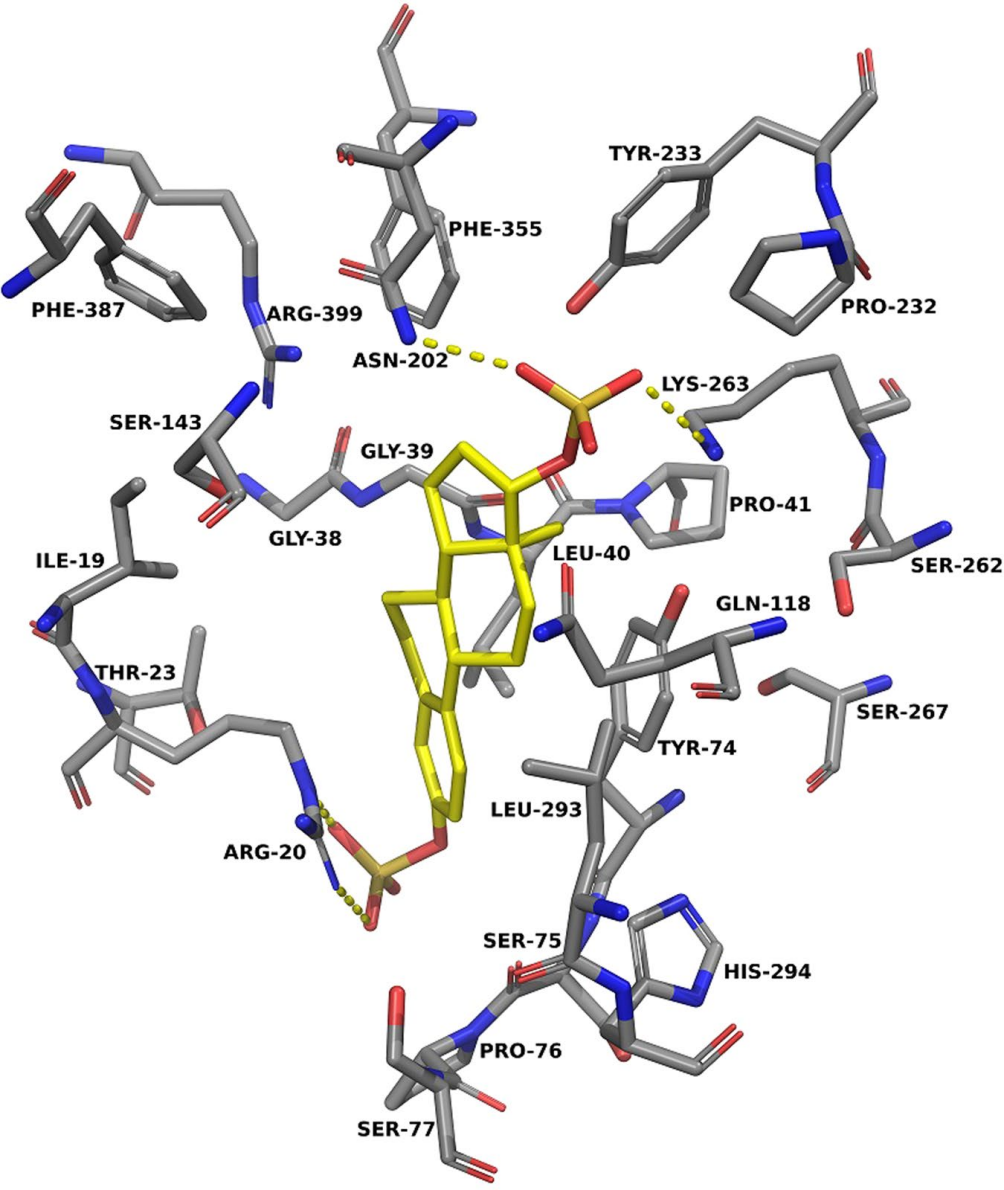
Estradiol disulfate docked into the active site of KAT-II. The amino acids with an atom within 5.0 Å of estradiol (yellow) were chosen for display. Residues Tyr-142 and Gly-144 were removed for clarity. The 3-sulfate group of estradiol disulfate forms hydrogen bonds (yellow dashes) with Arg-20, and the 17-sulfate group forms hydrogen bonds (yellow dashes) with Asn-202 and Lys-263. Image generated with PyMOL^67^.

The docking of estradiol 17-sulfate showed differences in that the 17-sulfate group is extended more towards the Lys-263 side-chain and forms a hydrogen bond with it (see Supplementary Fig. S6). Estradiol disulfate displayed a mix of features of all the estrogen compounds, with the 3-sulfate position hydrogen bonding with Arg-20 and the 17-sulfate position hydrogen bonding with the Asn-202 and Lys-263 residues (Fig. 7).

Experiments with phosphorylated estrogens have shown that adding a phosphate moiety onto the 17-position, in comparison to the 3-position, improves the potency of the molecule in inhibiting the PLP-dependent aspartate aminotransferase^41^. A similar result, displaying the importance of 17-position sulfate moieties, was also observed in the inhibition assays with the KAT enzymes. The docking suggesting that the 17-sulfate on estradiol disulfate and estradiol 17-sulfate binding to Lys-263 is in accordance with this. Lys-263 is a catalytically significant side-chain as it binds to PLP, which is important in the regular activity of KAT (Fig. 2). This, along with the hydrogen bonding with Asn-202 and Arg-20, suggests that estradiol disulfate interacts with many key residues in the active site that would normally interact with the substrate and the cofactor.

## Materials and Methods

### General Procedures

Commercially available reagents were used without additional purification unless otherwise stated. estradiol, estradiol 3-sulfate sodium salt, estradiol 3,17-disulfate dipotassium salt, estrone sulfate potassium salt were purchased from Sigma-Aldrich (Sydney, Australia). Methanol and dimethyl sulfoxide (DMSO) were purchased from Thermo Fisher Scientific (Scoresby, Australia). PBST (phosphate buffered saline, pH 7.4 with 0.05% Tween 20) was purchased from Sigma, and SPR reagents (40 mM EDC, 10 mM sulfo-NHS, 10 mM sodium acetate, 1 M ethanolamine HCl, pH 8.5) were purchased from Bio-Rad Laboratories (Gladesville, Australia).

SPR binding affinity measurements were performed using the Biacore T200 with a CM5 sensor chip (GE Healthcare Life Sciences, Sydney, Australia). The experiment was prepared and obtained with the Biacore T200 Control Software, and analysed with the Biacore T200 Evaluation Software.

GraphPad Prism v7.02 software^59^ was used to develop a nonlinear regression fit of the data collected in the inhibition assays. The IC_50_ is reported with the standard error.

### Protein Preparation

The expression and purification of recombinant KAT-I and KAT-II protein was completed as previously described^60,61^.

### HPLC Inhibition Studies Using Recombinant Human KAT

The KAT inhibition assay was based on that described previously^35^. 0.7 µg of KAT-I or KAT-II was incubated at 37 °C for 10 minutes in a 50 µL reaction mixture containing PLP (50 µM), α-ketoglutarate (5 mM), L-KYN (5 mM) in PBS, pH 7.4, and the inhibitor studied (1–2000 µM). Following incubation, the reaction was terminated by adding formic acid (0.8 M) in a 1:1 ratio. 50 µL of the mixture was transferred into a vial with 950 µL of water for HPLC analysis.

The production of KYNA was measured using HPLC with UV detection at a wavelength of 330 nM, using a C18 reverse-phase column, and 50% (v/v) water and 50% (v/v) methanol mobile phase. The data was collected in triplicate.

### Surface Plasmon Resonance Binding Assay

Prior to performing the SPR experiments, the solutions were filter sterilised for 10 min at 25 °C. KAT-II was diluted to a concentration of 350 µg/mL using sodium acetate (pH 4.5). The Biacore T200 compartment temperature was controlled at 25 °C and the flow rate was set to 10 µL/min. Using a 1:1 mixture of EDC (400 mM) and sulfo-NHS (100 mM), two flow cells on a CM5 sensor chip (containing a carboxymethylated dextran surface) were activated. Flow cell one contained a blank immobilisation (PBST buffer injection) and flow cell two had the prepared KAT-II sample injected. The two flow cells were deactivated using an injection of 1 M ethanolamine.

The analyte, estradiol disulfate, was prepared in running buffer (PBST with 5% DMSO) in a concentration series from 170 nM-43750 nM. The analytes were injected in a multicycle kinetics mode using a flow rate set to 30 µL/min, contact time of 90 seconds and a dissociation time of 300 seconds, with an extra 50% DMSO wash performed after each injection. Solvent correction was performed before and after the estradiol disulfate injections, and also every thirty cycles, to account for the use of DMSO in the analyte sample preparation and the running buffer. The data was collected in triplicate.

### Computational Docking of Estrogen Compounds at the Active Site of KAT-II

The human KAT-II crystal structure (PDB ID: 2R2N^62^) was downloaded from the Protein Data Bank. The PLP cofactor was removed as it is thought to compete with the ligands in the active site rather than being present simultaneously. The protein preparation wizard was used in Maestro (version 10.4.017) to optimise and minimise the protein, and hydrogen atoms were added and water molecules not participating in the reaction were removed. The ligands were prepared using LigPrep^63^, using the OPLS-2005 force field^64^. The active site was defined by the location of the existing (and natural substrate of KAT) ligand, kynurenine, in the PDB file. The ligands were then docked using the Glide docking program^65^, with XP (extra precision) docking^66^.

### Data Availability

The data generated and analysed during the current study is available in the Figshare repository (https://figshare.com/projects/Inhibition_of_human_kynurenine_aminotransferase_isozymes_by_estrogen_and_its_derivatives/24823).

## Conclusions

KYNA, a metabolite in the tryptophan metabolic pathway, is produced by the KAT enzymes, of which KAT-II is thought to be the isoform that produces the majority of KYNA in the human brain. KYNA is found elevated in the brain of patients with schizophrenia, and is therefore implicated in the condition. Low circulating estrogen levels are also associated with the onset and severity of patients with schizophrenia. The activity of the KAT enzymes has been previously studied with estrogens and estrogen conjugates to determine their inhibitory ability, but in rat homogenates only.

We have found estradiol disulfate to be the most potent amongst the group of derivatives inhibiting KAT activity, with some activity also displayed by estrone sulfate. Despite showing inhibition, estradiol and estradiol 3-sulfate were, relatively, the weaker inhibitors. In combination with molecular docking studies, this shows that increased potency for KAT-II inhibition for estrogens is mainly derived from the 17-sulfate moiety. This can also be exploited in the design of novel KAT-II inhibitors, and also contribute to the improvement of existing inhibitors.

Changes in estrogen levels induced by oral contraceptives, pregnancy and the menstrual cycle can influence the tryptophan metabolic pathway. Estrogens have the ability to be formed in the brain analogous to that of peripheral tissues, and have a similar capacity to being metabolised as a consequence of enzymatic activity in the brain. Owing to the inhibitory capability of the steroids, and particularly their sulfate derivatives, the distribution of the metabolic enzymes in the brain may dictate the potential for these compounds to inhibit the KAT enzymes. Consequently, this will affect the production of tryptophan metabolites, including that of KYNA, in the brain.

Ultimately, steroid dysfunction in schizophrenia may not be confined to estrogen dysfunction alone, and is it possible that many steroid hormones and their metabolites are involved. There are multiple pathways in which estrogen may be affecting the brain but a role involving the NMDA receptor antagonist KYNA is potentially a significant one, which may be consistent with the kynurenic acid hypothesis of schizophrenia as a lack of KAT inhibition by estrogen derivatives in the brain can cause an imbalanced oversupply of KYNA.

## Electronic supplementary material

Supplementary Information
