# Supplementary material for: Inhibition of human kynurenine aminotransferase isozymes by estrogen and its derivatives
Source: Sci Rep. 2017 Dec 14;7:17559. doi: 10.1038/s41598-017-17979-7 (PMC5730616; doi:10.1038/s41598-017-17979-7)
Supplement: Supplementary file 1 — Supplementary Information [file 41598_2017_17979_MOESM1_ESM.pdf]

Submission to: Scientific Reports

Inhibition of human kynurenine aminotransferase isozymes by estrogen and its derivatives

Gayan S. Jayawickrama[1], Alireza Nematollahi[1], Guanchen Sun[1], Mark D. Gorrell[2], W. Bret Church[1*]

[1]Group in Biomolecular Structure and Informatics, Faculty of Pharmacy, The University of Sydney, Sydney NSW 2006, Australia

[2]Molecular Hepatology Laboratory, Centenary Institute and Sydney Medical School, The University of Sydney, Sydney, NSW 2006, Australia

*To whom correspondence should be addressed:

e-mail address: bret.church@sydney.edu.au

Postal address: Faculty of Pharmacy A15, The University of Sydney, Sydney NSW 2006, Australia

Supplementary Data

Figure Legends

Figure S1. Estradiol disulfate interactions with KAT-II using SPR.

Steady state affinity model of estradiol disulfate interacting with KAT-II bound on a CM5 sensor chip at 25 °C (KD: 5.05×10^{-6}, chi^2: 0.481).

Figure S2. Estradiol docked into the active site of KAT-II.

The amino acids with an atom within 5.0 Å of estradiol (yellow) were chosen for display. Residues Tyr-142, Gly-144, Gln-289, and Val-290 were removed for clarity. The aromatic A ring of estradiol forms pi-pi interactions (green dashes) with Tyr-74, and pi-cation (blue dashes) interactions with Arg-20. The 3-hydroxyl group forms hydrogen bonds (yellow dashes) with Ser-75, and the 17-hydroxyl group forms hydrogen bonds with Arg-399. Image generated with PyMOL[63].

Figure S3. Estrone docked into the active site of KAT-II.

The amino acids with an atom within 5.0 Å of estradiol (yellow) were chosen for display. Residues Tyr-142, Gly-144, Gln-289, and Val-290 were removed for clarity. The aromatic A ring of estrone forms pi-pi interactions (green dashes) with Tyr-74, and pi-cation (blue dashes) interactions with Arg-20. The 3-hydroxyl forms hydrogen bonds (yellow dashes) with Ser-75, and the 17-carbonyl group forms hydrogen bonds with Arg-399 and Asn-202. Image generated with PyMOL[63].

Figure S4. Estradiol 3-sulfate docked into the active site of KAT-II.

The amino acids with an atom within 5.0 Å of estradiol (yellow) were chosen for display. Residues Tyr-142 and Gly-144 were removed for clarity. The 3-sulfate group of estradiol 3-sulfate forms hydrogen bonds (yellow dashes) with Arg-20. Image generated with PyMOL[63].

Figure S5. Estrone sulfate docked into the active site of KAT-II.

The amino acids with an atom within 5.0 Å of estradiol (yellow) were chosen for display. Residues Tyr-142 and Gly-144 were removed for clarity. The 3-sulfate group of estrone sulfate forms hydrogen bonds with Arg-20, and Thr-23. Image generated with PyMOL[63].

Figure S6. Estradiol 17-sulfate docked into the active site of KAT-II.

The amino acids with an atom within 5.0 Å of estradiol (yellow) were chosen for display. Residues Tyr-142, Gly-144, Gln-289, and Val-290 were removed for clarity. The 17-sulfate group of estradiol 17-sulfate forms hydrogen bonds (yellow dashes) with Lys-263. Image generated from PyMOL[63].

Figures

Figure S1. Estradiol disulfate interactions with KAT-II using SPR.

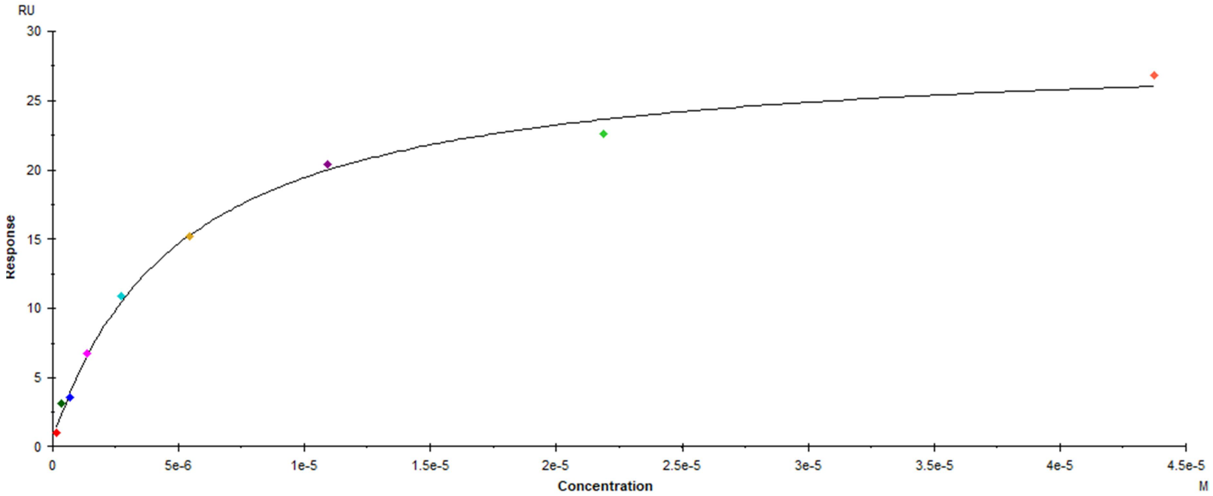

Figure S2. Estradiol docked into the active site of KAT-II.

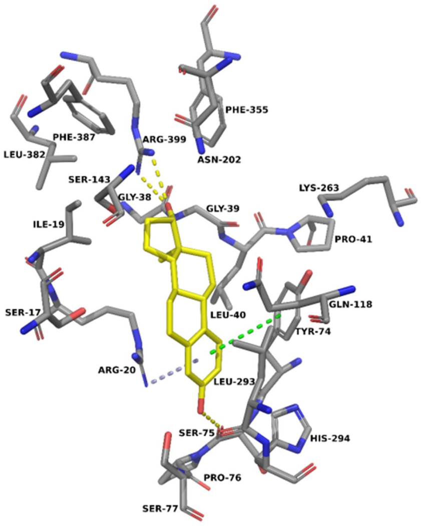

Figure S3. Estrone docked into the active site of KAT-II.

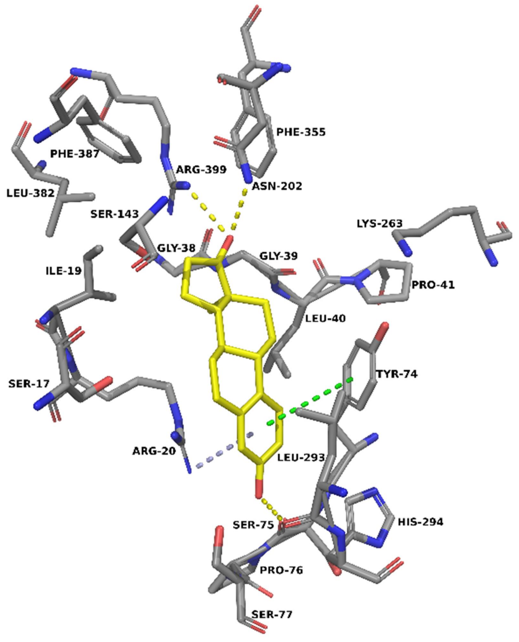

Figure S4. Estradiol 3-sulfate docked into the active site of KAT-II.

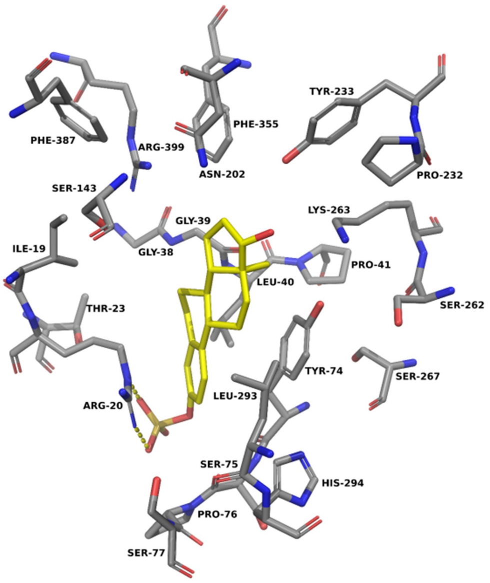

Figure S5. Estrone sulfate docked into the active site of KAT-II.

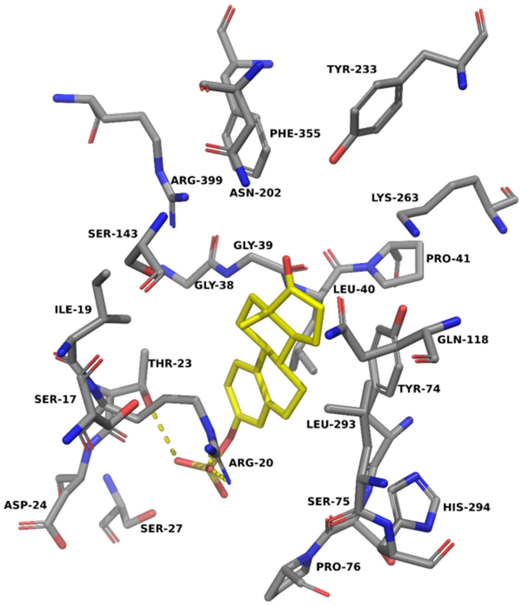

Figure S6. Estradiol 17-sulfate docked into the active site of KAT-II.

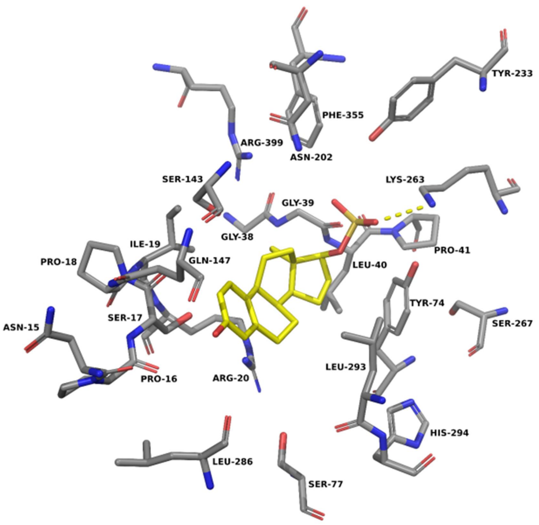

Tables

PLP Concentration (µM)	Estrogen Disulfate Inhibition % ± S.E.	PF-04859989 Inhibition % ± S.E.
10	90.78 ± 0.26	52.32 ± 1.12
50	85.16 ± 1.82	51.64 ± 2.04
100	78.83 ± 0.77	48.75 ± 0.81
200	76.28 ± 1.88	50.71 ± 0.68

Table S1. Inhibition of estrogen disulfate and PF-04859989 on KAT-II. The inhibition caused by estrogen disulfate lowers with increasing PLP concentrations in the assay, but remains relatively unchanged in the case of the irreversible inhibitor, PF-04859989.

Ligand	Glide XP Score (Kcal/mol)	Glide Energy (Kcal/mol)	Hydrogen Bonds	Ligand Interacting Atoms	Protein Interacting Atoms	Bond Length (Å)
Estradiol	-6.3	-37.0	3	H (3-OH)<br>O (17-OH)<br>O (17-OH)	O (Ser-75)<br>$H^{\eta12}$ (Arg-399)<br>$H^{\eta22}$ (Arg-399)	1.7<br>1.9<br>2.3
Estradiol 3-sulfate	-6.2	-30.3	2	O (3-SO_4)<br>O (3-SO_4)	$H^{\eta21}$ (Arg-20)<br>H^{ε} (Arg-20)	1.6<br>1.6
Estradiol disulfate	-8.0	-40.3	4	O (3-SO_4)<br>O (3-SO_4)<br>O (17-SO_4)<br>O (17-SO_4)	$H^{\eta21}$ (Arg-20)<br>H^{ε} (Arg-20)<br>$H^{\delta21}$ (Asn-202)<br>$H^{\zeta3}$ (Lys-263)	1.6<br>1.6<br>1.9<br>2.3
Estradiol 17-sulfate	-4.1	-36.6	1	O (17-SO_4)	$H^{\zeta3}$ (Lys-263)	2.5
Estrone	-7.4	-40.2	3	H (3-OH)<br>O (17=O)<br>O (17=O)	O (Ser-75)<br>$H^{\delta22}$ (Asn-202)<br>$H^{\eta12}$ (Arg-399)	2.3<br>2.2<br>2.1
Estrone sulfate	-5.2	-26.2	3	O (3-SO_4)<br>O (3-SO_4)<br>O (3-SO_4)	H^{ε} (Arg-20)<br>$H^{\eta21}$ (Arg-20)<br>$H^{\gamma1}$ Thr-23	1.9<br>2.3<br>1.9

Table S2. XP docking of estrogen compounds into KAT-II active site. Displayed are the Glide XP score, Glide energy, and number of hydrogen bonds formed between the ligand and the KAT-II protein with details on which atoms and residues take part in these hydrogen bonds.
